# Discovery of P1736, a Novel Antidiabetic Compound That Improves Peripheral Insulin Sensitivity in Mice Models

**DOI:** 10.1371/journal.pone.0077946

**Published:** 2013-10-23

**Authors:** Jessy Anthony, Aditya Kelkar, Chandan Wilankar, Vijayalakshmi Ranjith, Sujit Kaur Bhumra, Shivaprakash Mutt, Nabajyoti Deka, Hariharan Sivaramakrishnan, Somesh Sharma, Adaikalasamy Rosalind Marita

**Affiliations:** 1 Department of Pharmacology, NCE Division, Piramal Enterprises Ltd, Mumbai, India; 2 Department of Chemistry, NCE Division, Piramal Enterprises Ltd, Mumbai, India; 3 Haffkine Institute for Training, Research & Testing, Acharya Donde Marg, Parel, Mumbai, India; Stellenbosch University, South Africa

## Abstract

Insulin resistance is a characteristic feature of Type 2 diabetes. Insulin resistance has also been implicated in the pathogenesis of cardiovascular disease. Currently used thiazolidinedione (TZD) insulin sensitizers although effective, have adverse side effects of weight gain, fluid retention and heart failure. Using fat cell-based phenotypic drug discovery approach we identified P1736, a novel antidiabetic molecule that has completed Phase II clinical trials. The present study evaluated the *in vitro* and *in vivo* pharmacological properties of P1736. P1736 is a non-TZD and it did not activate human PPAR(Peroxisome Proliferator Activated Receptor Gamma )receptors. P1736 caused dose dependent increase in glucose uptake (EC_50_-400nM) in the insulin resistant 3T3 adipocytes. The compound (10µM) induced translocation of GLUT-4 (Glucose Transporter type 4) transporters in these adipocytes while metformin (1.0mM) was inactive. In diabetic *db/db* mice, P1736 (150mg/kg) was more efficacious than metformin in lowering plasma glucose (35% vs 25%) and triglyceride levels (38% vs 31%). P1736 tested at 5mg/kg, twice daily doses, reduced glucose by 41% and triglycerides by 32%, in *db/db* mice. These effects were not associated with adverse effects on body weight or liver function. Rosiglitazone (5mg/kg, twice daily) caused 60% and 40 % decreases in glucose and triglyceride levels, respectively. However, rosiglitazone induced 13% weight gain (p<0.05) in *db/db* mice. P1736 was also efficacious in *ob/ob* mice wherein 30-35% decrease in glucose and significant improvement in hyperinsulinemia were observed. Administration of P1736 to *ob/ob* mice resulted in 70% increase in glucose uptake in soleus muscles while metformin caused 38% increase. P1736 exhibited excellent safety profile and was weight neutral in all preclinical models of diabetes. Thus, P1736 with its unique pharmacology coupled with PPAR- independent mode of action could represent an alternative option in the management of insulin resistant Type 2 diabetic patients.

## Introduction

Insulin Resistance is a common feature of metabolic disorders such as obesity, cardiovascular disease and Type 2 Diabetes. Further, insulin resistance has been implicated in the pathogenesis of cardiovascular complications of diabetes [1,2]. Type 2 diabetic patients have 2-fold higher risk of mortality from heart disease compared to non-diabetic subjects. Currently available thiazolidinedione (TZD) drugs such as pioglitazone and rosiglitazone although potent [3] have been associated with several adverse events including increased cardiovascular mortality with rosiglitazone [4,5], liver toxicity with troglitazone [6], weight gain and fluid retention with all the TZDs. Most of the side effects of TZDs are ascribed to the activation of peroxisome proliferator activated receptor-gamma, PPARγ [3]. Thus, there exists an opportunity to develop novel and safe insulin sensitizers that act independent of PPAR activation. 

**Figure 1:**
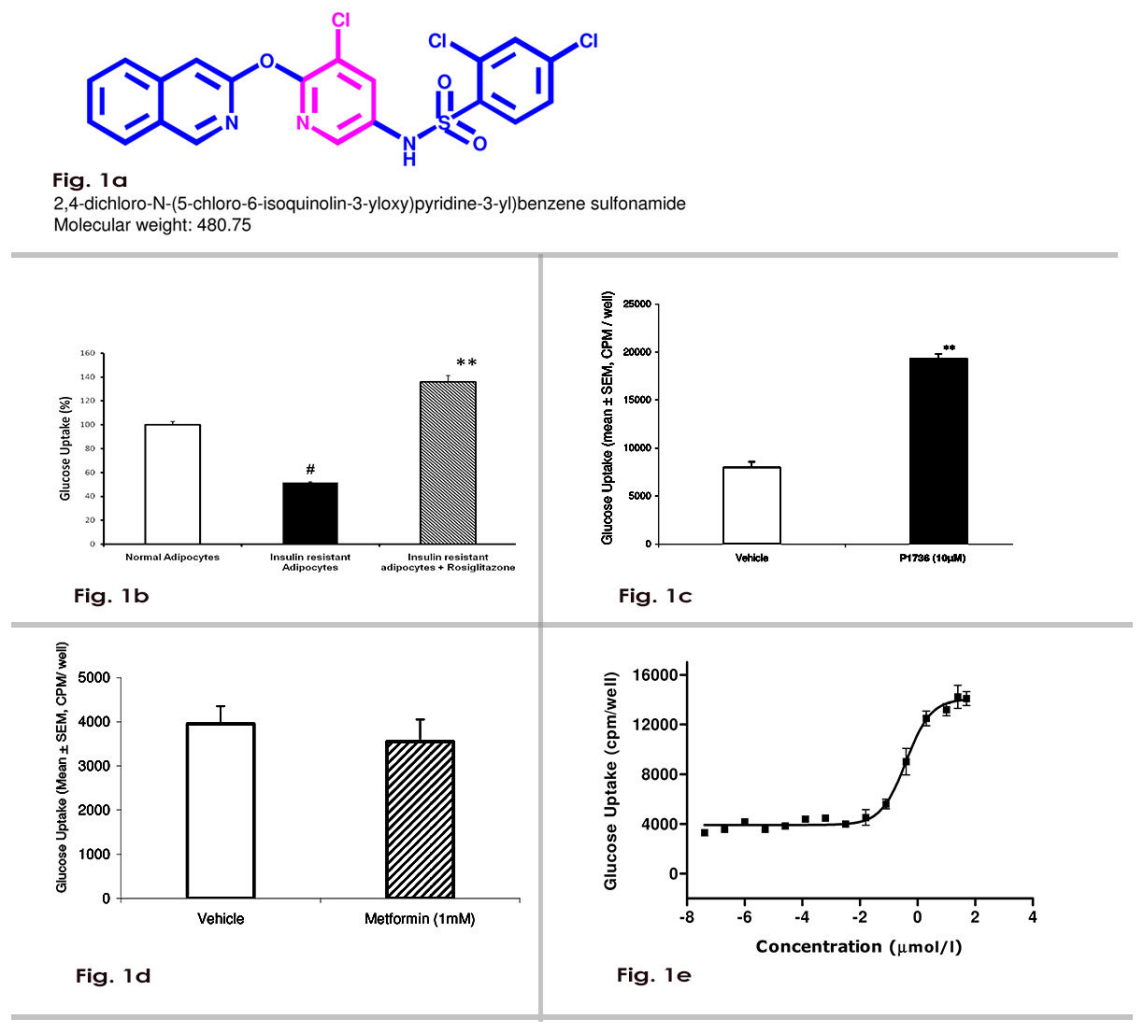
P1736 acts as a potent *in* *vitro* insulin sensitizer. **a**, Chemical structure of P1736. **b**, Insulin stimulated glucose uptake in normal adipocytes(empty bar), insulin resistant adipocytes(solid bar) and insulin resistant adipocytes treated with rosiglitazone (bar with stripes). **c**, Glucose uptake in the dexamethasone - induced insulin resistant adipocytes exposed to vehicle(empty bar) or P1736 (10 µM, solid bar). **d**, Glucose uptake in the dexamethasone-induced insulin resistant adipocytes exposed to vehicle (empty bar) or metformin (1.0mM, bar with stripes). **e**, Dose response of P1736 on glucose uptake in dexamethasone - induced insulin resistant adipocytes. Data are expressed as Mean ± SEM. Difference between vehicle and treatment groups were statistically evaluated by t-test., ^#^p<0.01 compared to normal adipocytes, **p<0.01 compared to insulin resistant adipocytes (vehicle).

In an effort to identify novel and safe insulin sensitizers, we employed a phenotypic drug discovery approach that uses fat-cell based screens that partly mirror insulin resistance seen in Type 2 diabetic patients. We used 3T3 adipocytes which were rendered insulin resistant by chronic exposure to dexamethasone. Dexamethasone-induced insulin resistance has been employed extensively in studies on insulin signaling [7,8]. We have previously shown that troglitazone, a clinically validated insulin sensitizer reversed dexamethasone-induced insulin resistance in 3T3 adipocytes [9] and the role of insulin signaling molecules in the development of insulin resistance has also been demonstrated [10]. Therefore, we hypothesized that the ability of small molecules to reverse the insulin-resistance, even partially, in a physiologically relevant cell type, could be a clinically relevant measure of insulin sensitivity [11]. Since P1736 is a non-thiazolidinedione (Figure 1a) and did not activate human PPAR, it would be interesting to know the pharmacological characteristics and the possible mode of action of the compound. The present study was carried out to evaluate the pharmacological characteristics of P1736 in preclinical models of diabetes.

## Experimental Procedures

### Materials

P1736 (free base, Batch 863-12B) and rosiglitazone maleate (Batch number AR-133-16) were synthesized in-house. 3T3-L1 cells were obtained from ATCC. Calf serum and fetal calf serum (FCS) were purchased from Hyclone (USA). Metformin hydrochloride, Dulbecco’s Modified Eagle’s Medium (DMEM), human insulin, bovine serum albumin (BSA), dexamethasone, 1-Methyl-3-isobutyl xanthine (IBMX), trypsin-EDTA, 4-(2-hydroxy ethyl)-1-piperazine ethane sulfonic acid (HEPES), penicillin G, streptomycin sulfate, cytochalasin B, 2-deoxy glucose, sodium dodecyl sulphate and wortmannin were obtained from Sigma Chemicals (USA). 2-deoxy-D-[^14^C]-glucose, C-14 labelled mannitol were from American Radiochemicals (USA). Polyclonal antibody against mouse GLUT-4 and FITC-labelled secondary antibody were from Abcam (USA). 

### Adipogenesis screen

Adipogenesis screening was carried out essentially according to the method of Choi et al [12]. Compounds from in-house chemical library were tested at a final concentration of 20 µg/ml using rosiglitazone (1.0 µM) as a positive control and mouse TNF-α (5ng/ml) as a negative control. Compounds that caused increase in adipogenesis by more than 5-fold of vehicle (0.1% DMSO) were considered positive and were selected for further screening.

### Screening in the insulin resistant model

Fully differentiated 3T3 adipocytes generated in 24-well plates were used after day 8 of differentiation. Insulin resistance was induced essentially as described before [9]. Briefly, adipocytes were incubated with 100 nM dexamethasone for six consecutive days. On day 2, compounds (10 µM) or rosiglitazone (0.1 µM) or metformin (1.0mM) were added to respective wells, in triplicate, and incubation was continued for 4 days with media changes every 48 h. For the dose response study, concentrations used were 4.1 x 10^-14^ M to 5 x 10^-5^ M. At the end of 4 days of exposure to compounds, the plates were processed for measurement of glucose uptake.

### Glucose uptake

Glucose uptake was measured essentially according to the method of Gao et al [13] using ^14^C-labeled 2-deoxy glucose as the tracer. Activity in this assay is defined as a statistically significant increase in glucose uptake of minimum 50% compared to the uptake seen in cells exposed to vehicle. Effect of wortmannin on glucose uptake was studied according to the method of Hausdorff et al [14] using 100 nM wortmannin. 

### GLUT-4 translocation

GLUT-4 translocation was evaluated by the method of Hausdorff et al [14] with a few modifications. Insulin resistant 3T3 adipocytes were prepared in 24-well plates fitted with coverslip inserts. After incubation with compounds, the media was removed. The cells were stimulated with 200nM insulin and processed for immunofluorescence detection using anti-GLUT-4 antibody (1:1000) followed by FITC labeled secondary antibody (1:1000). Images were obtained using BioRad 1024 Laser Scanning Confocal Microscope. 

### Miscellaneous assays

Transactivation activity against human PPARγ and PPARα receptors was determined using luciferase reporter gene assays in the Laboratory of CloneGen Biotechnology, Michigan, USA. Activities of protein tyrosine phosphatase-1b (PTP-1B) and adenosine mono phosphate activated kinase (AMPK) were evaluated at MDS Pharma, Korea. Evaluation of activity against human 11<beta>-Hydroxysteroid dehydrogenase-1 was outsourced to University of Edinburg, UK which used a cell based method [15].

### Animal studies

Eight week old male *ob/ob* mice and 5-8 week old male *db/db* mice were purchased from Jackson Laboratories (USA). Male *db/db* mice of 5-6 weeks age and 6 weeks old Wistar rats from the animal facility of Piramal Enterprises Limited were also used for some experiments. Animals were housed in a temperature-controlled (24°C) facility with a 12-hour light/dark cycle and had free access to feed (D12450B, Research Diets, USA) and water. All the animal experiments were carried out according to the guidelines of CPCSEA (Committee for the Purpose of Control and Supervision Of Experiments on Animals; www.cpcsea.com) and the Institutional animal ethics committee (IAEC- Reg no. 29/1999 CPCSEA) of Piramal Enterprises approved all experimental procedures.

### Evaluation of antidiabetic activity in db/db mice

After two weeks of acclimatization, *db/db* mice were deprived of food for 4h. Their weights were measured and blood was obtained by retro-orbital puncture for the determination of plasma glucose. The animals were randomized into groups (7-10/group) based on body weight and plasma glucose levels. Animals were dosed orally once a day, between 9.00 -10.00am for 15 consecutive days, with either P1736 (150mg/kg) or metformin (150mg/kg) or 0.5% carboxy methyl cellulose (CMC) vehicle. The dose of 150 mg/kg was chosen for once-a-day dosing of P1736, on the basis of its pharmacokinetic profile and to maintain high plasma concentrations. Body weight was measured daily. On days 10 and 15, blood samples were collected at the end of the 4h fasting after drug administration. Plasma samples were analysed for biochemical parameters, by enzymatic methods using autoanalyser 902(Hitachi instruments, Japan). Plasma adiponectin levels were measured using mouse ELISA kits (Millipore Inc. USA).

For the dose response experiment, groups of male *db/db* mice (8/ group) received twice daily doses of P1736 (5-, 25-, 50-, 100- and 200 mg/kg) or rosiglitazone (5 mg/kg) in 0.5% CMC vehicle. The doses were given during 8.00-9.00 am and 5.00-6.00 pm daily. On day 15, blood was collected and plasma samples were analysed as described above.

### Evaluation of antidiabetic activity in ob/ob mice

After one week of acclimatization, *ob/ob* mice (8/group) were dosed orally, twice daily for 10 consecutive days, with either P1736 (100 or 200 mg/kg) or vehicle. Rest of the procedure is exactly the same as that used for *db/db* mice and as described above.

### Studies on *ex vivo* muscle glucose uptake in *ob/ob* mice

Groups of *ob/ob* mice (10/group) were administered twice daily doses of P1736 (1- or 5 mg/kg) or rosiglitazone (5 mg/kg) in 0.5% CMC vehicle for 13 consecutive days. On day 9, food was withheld for 4 h and blood was collected for the analysis of plasma chemistry and plasma insulin levels. On days 12 and 13, 3-4 animals from each group were sacrificed and soleus muscles were excised. Radiolabelled glucose uptake was measured in the isolated soleus muscles by the method of Zierath and colleagues[16]. Plasma Insulin was estimated using mouse ELISA kit (Linco, USA)

### Evaluation of plasma volume in rats

Groups of male and female Wistar rats were administered P1736 (100 mg/kg and 300 mg/kg) or rosiglitazone (40 mg/kg) in 0.5% CMC vehicle once daily for 28 consecutive days. Rats were weighed every week. At the end of 28 days, plasma volume was measured in anaesthetized rats using Evans Blue Dye method [17].

### Statistical analysis

Data are expressed as Mean ± SEM. Statistical analysis were performed using Student ‘t’ test for comparisons between vehicle and compound or standard. Significant differences were identified at p<0.05. Dose response data were analysed by Non-linear Curve fitting program. All the statistical analyses were carried out using the GraphPad Prism software (Version 3.03). 

## Results

### Identification of P1736 as an *in vitro* insulin sensitizer

We screened 1558 compounds in adipogenesis assay. In this assay the vehicle induced 7-10% adipogenesis. Rosiglitazone (1.0 µM) caused >90% adipogenesis and is considered positive. Mouse TNF-α used as a negative control caused 1-2% adipogenesis. Of the 1558 compounds tested, 142 were positive representing a hit rate of 9.1%. P1736 (43 µM) was positive in adipogenesis screen.

Positives from the adipogenesis screen were tested in insulin resistant adipocytes to select compounds that promote glucose uptake, a functional endpoint of insulin action. Insulin caused 7-10 fold increase (from basal) in glucose uptake in normal (insulin sensitive) adipocytes. In dexamethasone-treated insulin resistant adipocytes, insulin caused only 3-4 fold increase in glucose uptake. This amounted to about 50% reduction (p<0.01) in glucose uptake compared to normal adipocytes (Figure 1b). As expected rosiglitazone, potentiated glucose uptake and re-sensitized the cells (Figure 1b). Among the 142 tested, 7 compounds caused significant increase in glucose uptake and these 7 were considered hits for further development. 

Exposure of insulin resistant adipocytes to P1736 (10 µM) resulted in increased glucose uptake by more than 2-fold compared to insulin resistant vehicle and induced re-sensitization (Figure 1c). Under identical condition, metformin (1.0 mM) treatment had no effect (Figure 1d) on glucose uptake. Further, P1736 caused dose dependent increase in glucose uptake in the insulin resistant adipocytes with EC_50_ of 0.40 µM (Figure 1e). The TZD, rosiglitazone exhibited an EC_50_ of 0.02 µM in this assay(data not shown).

### P1736 does not cause transactivation of human PPAR

**Figure 2:**
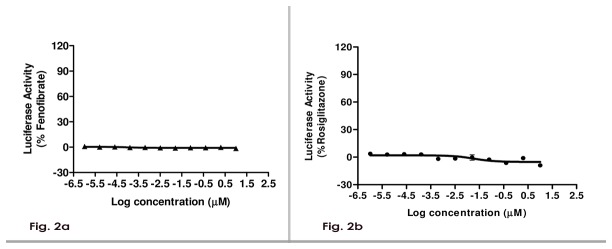
P1736 does not activate human Peroxisome Proliferator Activated Receptors (PPAR). **a**, P1736 was added to HEK-293 cells expressing ligand binding domain of human PPARα receptor and the transactivation ability was assayed using renilla luciferase activity. Fenofibrate was used as the positive control. **b**, P1736 was added to HEK-293 cells expressing ligand binding domain of human PPARγ receptor and the transactivation ability was assayed using rosiglitazone as the positive control.

As depicted in Figure 2a, P1736 did not activate PPARα receptor upto 10 μM in cell based transactivation assay. Fenofibrate a clinically validated hypolipidemic agent and a PPARα agonist caused significant activation in the assay. In assays measuring the transactivation ability towards PPARγ isoform of the receptor, P1736 had no significant transactivation ability upto 10 μM (Figure 2b) while rosiglitazone, a strong agonist induced marked activation.

### P1736 reduces circulating glucose and triglyceride levels in *db/db* mice

**Figure 3:**
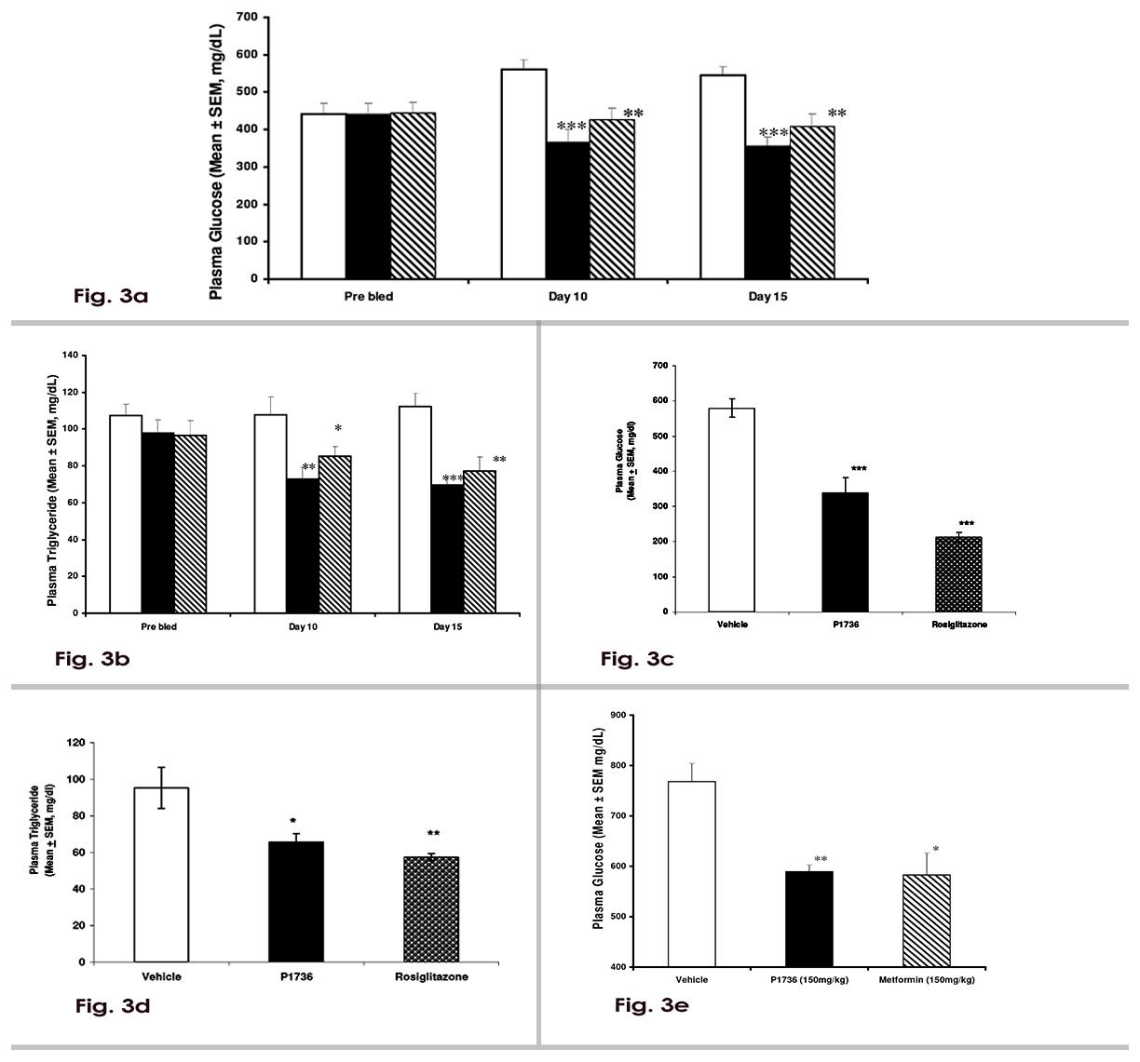
P1736 reduces circulating glucose and triglyceride levels in diabetic *db/db*mice. **a**, Plasma glucose levels in male *db/db* mice that received once daily oral doses of vehicle(empty bar) or P1736 (150 mg/kg, solid bar) or metformin (150 mg/kg, bar with stripes) for 15 days. **b**, Plasma triglyceride levels of *db/db* mice that received vehicle(empty bar) or P1736 (150 mg/kg, solid bar) or metformin (150 mg/kg, bar with stripes) for 15 days. **c**, Plasma glucose and **d**, Plasma triglyceride levels of male *db/db* mice that received vehicle(empty bar) or P1736 (5 mg/kg twice daily, solid bars) or equal dose of rosiglitazone (bar with dots). **e**, Plasma glucose levels of male *db/db* mice with high initial plasma glucose, that received vehicle(empty bar) orP1736 (150 mg/kg once daily, solid bar) or metformin (150 mg/kg once daily, bar with stripes) for 10 days. Data are expressed as Mean ± SEM for 7 - 10 animals per treatment group. Differences between vehicle and compound treated groups were statistically evaluated by t-test. *p<0.05, **p<0.01, ***p<0.001 vs.vehicle.

Treatment of *db/db* mice with P1736 (150 mg/kg) caused 35% (p<0.001) reduction in plasma glucose levels, both on day 10 and day 15 (Figure 3a). Metformin tested at the same dose caused 24% (p<0.01) and 25% (p< 0.01) reductions in plasma glucose, on day 10 and day 15, respectively (Figure 3a). P1736 caused 30% decrease (p< 0.01) in plasma triglyceride levels that was apparent on day 10 and the effect persisted till the end of the dosing period when a 38% decrease was observed (Figure 3b). Metformin caused 18% (p<0.05) and 31% (p<0.01) decreases in triglyceride levels on day 10 and day 15, respectively. In this experiment P1736 was more efficacious in lowering plasma glucose and triglyceride levels, than metformin. In *db/db* mice, P1736 caused significant increase in plasma adiponectin levels from 7.5 ± 0.44 µg/ml to 12.4±0.33µg/ml (p<0.001) while metformin treatment did not affect the adiponectin levels(8.9±0.64µg/ml) significantly.

To determine the dose response effect of P1736 in *db/db* mice, an experiment using 5 mg/kg to 200 mg/kg of the compound was carried out. In this experiment we could not establish EC_50_ since all the doses reduced plasma glucose and triglyceride levels (data not shown). However, at the lowest tested dose of 5 mg/kg, twice daily, P1736 induced 40% (p<0.001) decrease in plasma glucose (Figure 3c) and 32% (p<0.05) decrease in triglyceride levels (Figure 3d). In this experiment, rosiglitazone caused about 60% reduction in plasma glucose levels (Figure 3c) and 40% reduction in plasma triglyceride levels (Figure 3d). 

P1736 (150 mg/kg) was able to attenuate high initial plasma glucose levels of *db/db* mice by 23% (p< 0.01, Figure 3e). Metformin (150 mg/kg) treatment caused a 25% (p< 0.05) reduction (Figure 3e) in the same experiment. 

### P1736 lowers plasma glucose and insulin levels in *ob/ob* mice

**Figure 4:**
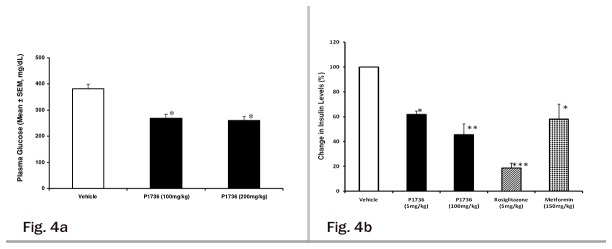
P1736 lowers plasma glucose and insulin levels in insulin resistant and obese *ob/ob* mice. **a**, Plasma glucose levels of male *ob/ob* mice that received oral doses of either vehicle (empty bar) or P1736 (100 mg/kg, 200 mg/kg, solid bars) measured after 10 days. **b**, Change in plasma insulin levels of *ob/ob* mice which received vehicle(empty bar) or P1736 (5 mg/kg, 100 mg/kg, solid bars) or rosiglitazone (5 mg/kg, bar with stripes) or metformin (150 mg/kg, dotted bars) for 10 days. Data are expressed in Mean ± SEM for 8 -12 animals per treatment group. Differences between vehicle and treatment groups were statistically evaluated by t-test *p<0.05, **p<0.01, ***p<0.001 vs. vehicle.

We evaluated the anti-diabetic and insulin sensitizing effects of P1736 in obese and insulin resistant *ob/ob* mice. P1736 was efficacious in reducing plasma glucose levels by 30% and 32% at doses of 100 and 200 mg/kg, respectively (Figure 4a). As illustrated in Figure 4b administration of P1736 to *ob/ob* mice induced significant lowering of plasma insulin levels, of 40% (p<0.05) and 50% (p<0.01) at 5 mg/kg and 100 mg/kg, respectively. Metformin (150 mg/kg) induced 43% (p<0.05) reduction while rosiglitazone caused 80% reduction in insulin (Figure 4b) levels in *ob/ob* mice.

### P1736 potentiates muscle glucose uptake in *ob/ob* mice

Improvement of hyperinsulinemia induced by P1736, prompted us to evaluate its effects on glucose uptake, in soleus muscles isolated from *ob/ob* mice after chronic administration. P1736 caused 70% and 67% increases in glucose uptake into soleus muscles, at 1.0 mg/kg and 5.0 mg/kg doses, respectively (Table 1). This effect was associated with concomitant plasma glucose lowering of 34% and 35%, respectively. Metformin (150 mg/kg) induced 38% increase in the glucose uptake and exhibited similar glucose lowering effect as that of P1736 (37% and 35%). The PPARγ agonist, rosiglitazone caused 147% increase in glucose uptake (Table 1) and 49% reduction in plasma glucose. 

**Table 1:** Effect of P1736 on Plasma Glucose and Glucose Uptake in Isolated Soleus Muscles of *ob/ob* mice.

**Compound (mg/kg)**	**Glucose Uptake in soleus muscle (% Increase)**	**Plasma Glucose (% Reduction)**
**P1736-05 (1.0)**	70*	**34**
**P1736-05 (5.0)**	67*	**35**
**Metformin (150.0)**	38*	**37**
**Rosiglitazone (5.0)**	147**	**49**

* p<0.05, **p<0.01 compared to vehicle

### Mode of action of P1736 as an insulin sensitizer

**Figure 5:**
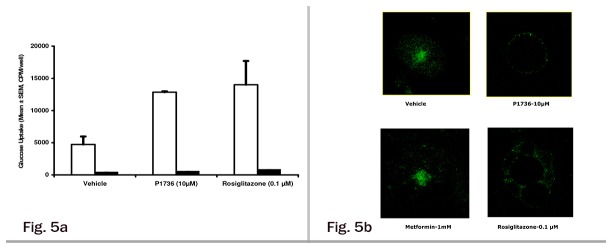
P1736 promotes glucose uptake by modulating insulin signaling pathways. **a**, Effect of P1736 on PI3 - Kinase mediated glucose uptake in insulin resistant 3T3 adipocytes. Insulin resistant adipocytes were treated with P1736 (10 μM) or rosiglitazone (0.1 μM) as mentioned in methods. At the end of 4 days, cells were treated with vehicle (empty bar) or 100 nM wortmannin (solid bar) for 30 minutes. Later 200nmol/l insulin stimulated 2-deoxyglucose uptake was determined. **b**, Effect of P1736 on GLUT-4 translocation in insulin resistant adipocytes. Insulin resistant 3T3 adipocytes were treated with vehicle or P1736 (10 μM) or Metformin (1 mM) or rosiglitazone (0.1 μM) for 4 days. Later the cells were stimulated with 200nmol/l insulin for 25 minutes. Whole cell immunofluorescence assay for GLUT-4 translocation was performed using rabbit antibody against GLUT - 4 followed by incubation with FITC labeled secondary antibody. Confocal images were obtained using Bio-Rad Laser Scanning Microscope.

In the insulin resistant adipocytes, stimulation of glucose uptake induced by P1736 was abolished by pretreatment with wortmannin (Figure 5a), a known inhibitor of PI3K (phosphatidylinositol 3-kinase) . Further, exposure of insulin resistant adipocytes to P1736 (10µM) resulted in complete translocation of GLUT-4 to plasma membrane as depicted in Figure 5b. In the same study, metformin (1 mM) was inactive while rosiglitazone (0.1 µM) also caused GLUT-4 translocation (Figure 5b). 

When P1736 was screened against a panel of membrane receptors, ion channels and enzymes no significant activity was detected against any target including PTP-1B and AMPK. The only positive hit was an inhibitory activity of 50% towards human 11<beta>-Hydroxysteroid dehydrogenase-1 enzyme.

### P1736 did not adversely affect body weight and liver function and plasma volume in rodents

**Figure 6:**
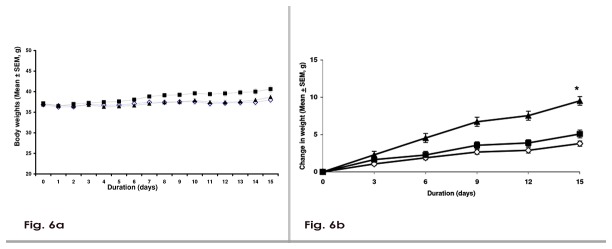
P1736 did not adversely affect body weight in preclinical diabetic models. **a**, Body weights of male *db/db* mice treated with vehicle (empty diamonds) or P1736 (150mg/kg, solid square) or metformin (solid triangle) for 15 days. Body weight was measured daily and recorded. **b**, Change in the weights of *db/db* mice treated with vehicle(empty diamonds) or P1736 (5 mg/kg twice daily, solid squares) or rosiglitazone (5 mg/kg, solid triangles) for 15 days. Data are expressed as Mean ± SEM for 7 - 10 animals per treatment group. * p< 0.05 compared to vehicle.

Treatment of *db/db* mice with 150 mg/kg of P1736 for 15 days did not affect body weight (Figure 6a) and exhibited a trend similar to the effect of metformin tested at the same dose. These body weight measurements were made during the efficacy evaluation in *db/db* mice model as illustrated in Figures 3a and 3b. Further, P1736 treatment of *db/db* mice was well tolerated without any evidence of liver toxicity or liver enzyme elevation (Table 2). At the lowest efficacious dose of 5mg/kg twice daily, tested in *db/db* mice for efficacy described in Figure 3c and 3d, P1736 did not exhibit any tendency towards weight gain while the TZD, rosiglitazone at the same dose induced 13% weight gain (p<0.05) during the experiment (Figure 6b). Similarly P1736 upto 200 mg/kg (twice daily) did not induce adverse changes in body weight (data not shown) or liver function (Table 3) in *ob/ob* mice model. 

**Table 2:** Effect of P1736 on Liver safety parameters in *db/db* mice.

**Treatment**	**Liver Weight (gm**)	**Plasma ALT (U/L**)	**Plasma AST (U/L**)
**Vehicle**	**1.8 + 0.10**	**91.2 + 12.7**	**100.6 + 8.8**
**P1736 (150mg/kg**)	**2.1+ 0.10**	**99.0 + 8.5**	**84.6 + 2.6**
**Metformin (150mg/kg**)	**2.1 + 0.08**	**75.3 + 6.4**	**101.7 + 8.4**

**Table 3:** Effect of P1736 on Liver safety parameters in *ob/ob* mice.

**Treatment**	**Plasma AST(U/L**)	**Plasma ALT(U/L**)	**Liver weight (gm**)
**Vehicle**	**468 ± 38**	**740 ± 71**	**4.3 ± 0.2**
**P1736 (100mg/kg**)	**547 ± 50**	**857 ± 72**	**4.6 ± 0.2**
**P1736 (200mg/kg**)	**470 ± 57**	**668 ± 92**	**4.4 ± 0.3**

Oral administration of P1736 to normal Wistar rats for 28 days did not cause significant increase in plasma volume or body weights in either male or female animals. The mean normalized plasma volume of male and female control rats were 33.44 ± 1.52 ml/kg and 35.56 ± 1.46 ml/kg, respectively. Plasma volumes of male and female rats administered 100 mg/kg of P1736 were 31.55 ± 0.27 ml/kg and 30.91 ± 0.03 ml/kg, respectively. The plasma volume of animals that received 300 mg/kg were 29.15 ± 0.02 and 33.02 ± 0.28 ml/kg, respectively. Rosiglitazone (40 mg/kg) treatment induced a significant (p<0.05) increase in plasma volume to 42.06 ± 1.39 ml/kg and 41.86 ± 1.52 ml/kg, in male and female rats, respectively.

## Discussion

The present pharmacological studies reveal that P1736: 1) is a non-thiazolidinedione insulin sensitizer with no potential to activate human PPAR receptors; 2) reduced plasma glucose and triglyceride levels in diabetic *db/db* mice model; 3) reduced plasma glucose levels in the obese and diabetic *ob/ob* mice; 4) its antidiabetic effect in *ob/ob* mice was associated with increased glucose uptake in the muscles and reduced plasma insulin levels; 5) The pharmacological effects of P1736 in preclinical models of diabetes were not associated with adverse effects on weight or liver safety profile. Further, the compound did not induce adverse effects on weight and plasma volume in normal Wistar rats after 28 daily doses; 6) *in vitro* studies revealed potentiation of GLUT-4 translocation and inhibition of human 11<beta>-Hydroxysteroid dehydrogenase-1 enzyme as likely mode of action of P1736.

We used phenotypic screening strategy that relied on cell-based assays rather than the conventional target-based approach in the early drug discovery. The novelty in the present study is the use of insulin resistant adipocytes, to select insulin sensitizers. Defective insulin-induced glucose uptake is one of the earliest abnormalities in Type 2 diabetic patients [18]. Adipose tissue exhibits reduced GLUT-4 transporters and impaired glucose metabolism in diabetic state. Further, adipose tissue-specific GLUT-4 knockout mice develop diabetes and insulin resistance [19]. Adipose tissue releases inflammatory cytokines and free fatty acids which impair insulin induced glucose uptake in other tissues. Therefore, we reasoned that compounds that increase glucose uptake of insulin resistant adipocytes, could correct this fundamental defect in diabetic patients.

3T3 adipocytes have been used in the discovery of diabetes drugs in the past. Recently, Zhang and colleagues [20] reported identification of rutamarin using 3T3 adipocytes. Choi and co-workers [12] have also used 3T3 adipocytes to identify insulin sensitizers. The difference between these previous studies and our study which used insulin resistant adipocytes is important from a clinical viewpoint. Insulin resistant Type 2 diabetic patients have 2-fold higher risk of cardiovascular disease. Therefore, it would be useful to develop compounds that would benefit insulin resistant patients and also serve as an alternative to TZD insulin sensitizers. 

Use of PPARγ agonists for the management of Type 2 diabetic patients has been intensely debated recently. With the restricted availability of rosiglitazone, pioglitazone is the only insulin sensitizer available for treating obese and insulin resistant diabetic patients. Partial agonists of PPARγ receptor and various kinds of modified PPARγ ligands are also under development [21]. Screening of our proprietary chemical library yielded 7 hits. These seven were again screened through several selectivity assays and pharmacokinetic effects to choose P1736 as the lead molecule. 

P1736 was effective in causing dose related potentiation of glucose uptake in the insulin resistant adipocytes with an EC_50_of 400nM while there was no effect on PPAR transactivation upto 25 times (10 µM) the concentration which promoted glucose uptake. Even though P1736 is weaker than rosiglitazone (EC_50_ - 20nM) in the insulin resistant adipocytes, it is quite remarkable for a compound that has no liability due to PPAR activation. Further, the *in vitro* effect on insulin signaling pathway including PI-3 kinase and GLUT-4 translocation are favourable characteristics needed in an antidiabetic drug. Our data on P1736 are in line with the recent report of rutamarin, which was identified using a screen involving GLUT-4 [20]. In our *in vitro* assays, metformin upto 1.0 mM concentration did not affect either glucose uptake or GLUT-4 translocation. Thus the *in vitro* properties of the compound appear to be different from rosiglitazone, a known PPARγ agonist as well as metformin which was inactive in our adipocyte model.

In obese and diabetic *db/db* mice, P1736 was more efficacious than metformin in lowering plasma glucose and triglyceride levels. This could be partly related to their *in vitro* insulin sensitizing effects seen in the insulin resistant adipocytes, among others. Since *db/db* mice used in this experiment were 7-week old and obese, hyperglycemia and dyslipidemia are a result of peripheral, mainly adipose tissue insulin resistance. Insulin sensitizing effect of P1736 on adipocytes and on GLUT-4 transporters, compared to lack of effect of metformin, support this contention. Hypertriglyceridemia is a known risk factor for cardiovascular disease in diabetic patients [22].The triglyceride lowering effect of P1736, in addition to its glucose lowering action seen in *db/db* mice, if translated in the clinic, would be helpful in improving cardiovascular complications through reduced triglyceride levels. Although we have not measured inflammatory cytokine levels in our study, we observed increased levels of adiponectin, an anti-inflammatory cytokine in *db/db* mice treated with P1736, but not with metformin. This could also contribute to the antidiabetic effects of P1736 in *db/db* mice.

 At the lowest tested dose of 5 mg/kg, P1736 caused robust glucose (40%) and triglyceride lowering (32%) in *db/db* mice although this was lower than the effects induced by rosiglitazone (60% and 40%) tested at the same dose. However lack of weight gain seen in *db/db* mice treated with P1736 (Figure 6b) contrary to significant increase of 13% (p<0.05) induced by rosiglitazone, is an added advantage for the compound.

P1736 was able to attenuate hyperglycemia in *db/db* mice exhibiting very high blood glucose levels (Figure 3e). Diabetes in *db/db* mice is progressive with old animals exhibiting insulin deficiency and severe hyperglycemia and younger ones exhibiting insulin resistance and hyperinsulinemia. Aged *db/db* mice exhibit β-cell failure; a condition that resembles Type 2 diabetic patients [23]. The source of hyperglycemia in these mice is hepatic glucose production. The mode of action of P1736 in this experiment involving severely diabetic *db/db* mice could partly involve inhibition of hepatic glucose production similar to the action of metformin, an established hepatic insulin sensitizer [24].

In *ob/ob* mice, a relatively low dose of P1736 (1-5 mg/kg) was able to lower plasma glucose and insulin levels to the same extent as metformin (150mg/kg). Hyperinsulinemia is a surrogate marker of peripheral tissue insulin resistance and often used as a biomarker in clinical trials. Further, hyperinsulinemia is considered as an independent risk factor for heart disease [25]. Therefore, reduction of hyperinsulinemia by P1736 is an indirect evidence of improvement in peripheral insulin sensitivity in *ob/ob* mice. Direct evidence for the insulin sensitising action of P1736 comes from the marked effects of P1736 on glucose uptake into soleus muscles of *ob/ob* mice. Thus, in this animal model of obesity and insulin resistance, P1736 improved both hyperinsulinemia and muscle insulin sensitivity while lowering plasma glucose levels.

The exact mechanisms mediating the anti-diabetic efficacy of P1736 in preclinical animal models of diabetes remain to be elucidated. However, in *vitro* studies indicate that P1736 upto a concentration of 10 µM does not activate nuclear receptors, PPARγ and PPARα, at concentrations 25-fold higher than EC_50_ required for potentiation of glucose uptake. Therefore, P1736 is unlikely to act through the nuclear receptors. This notion is supported by the lack of an effect of P1736 on plasma volume in rats in experiments in which the TZD insulin sensitizer, rosiglitazone induced a significant increase. Further, in all the pharmacological studies conducted, P1736 was weight neutral and did not adversely affect liver function tests suggestive of PPAR –independent action. 

Our target exploration studies, reveal that P1736 inhibited human 11<beta>-Hydroxysteroiddehydrogenase-1 enzyme. Adipose tissue and liver of diabetic animals express high levels of 11<beta>-Hydroxysteroid dehydrogenase-1 [26,27]. It is possible that P1736 elicits its anti-diabetic action partly by inhibiting 11<beta>-Hydroxysteroid dehydrogenase-1 in the liver or adipose tissues of these diabetic animal models. Further, P1736 could act as a non-steroidal glucocorticoid receptor antagonist, in diabetic animals attenuating metabolic abnormalities driven by glucocorticoid excess. This is supported by the *in vitro* insulin sensitizing effect of the steroid RU486, a non-specific glucocorticoid antagonist in dexamethasone treated adipocytes [9]. While the manuscript was under preparation, we came across a report by Konstantinapoulos and co-workers [28] on the identification of methazolamide, using TNFα-induced insulin resistance and following gene expression signature [29]. In contrast to this we followed glucose uptake, a clinically useful end point of insulin action. It is known that both dexamethasone and TNF-α induce insulin resistance by promoting oxidative stress [30]. Antioxidant, manganese (iii) tetrakis (4-benzoic acid) porphyrin, MnTBAP has been reported to reduce glucose levels in *ob/ob* mice [30]. Thus, it is possible that P1736 could also act as an antioxidant in *ob/ob* mice to cause reduction in plasma glucose levels.

One limitation of our phenotypic screening approach, as expected from any cell-based approach, is the low throughput that could limit its wider pharmaceutical application. Studies are in progress to modify the approach without compromising on the sensitivity. We have evaluated the efficacy of P1736 in genetic models of Type 2 diabetes such as *ob/ob* and *db/db* mice which do not completely mimic the metabolic characteristics of patients. Studies using diet-induced-obese mice are currently ongoing. We have not evaluated the effect of P1736 in normal animals which could throw light on the effect of P1736 on oral glucose handling.

Further, P1736 did not cause hypoglycemia in treated animals, confirming its mode of action as an insulin sensitizer. The extra ordinary safety of P1736 coupled with interesting pharmacological properties, provided us with a unique molecule with all the desirable properties required for clinical development. 
