# Correction: Discovery of P1736, a Novel Antidiabetic Compound That Improves Peripheral Insulin Sensitivity in Mice Models

**DOI:** 10.1371/journal.pone.0091390

**Published:** 2014-02-28

**Authors:** The PLOS ONE Staff

The name of the sixth author was given incorrectly. The correct name is: Shivaprakash Jagalur Mutt. The correct abbreviation in the Author Contributions Statement is: SJM.
