# Correction: Discovery of P1736, a Novel Antidiabetic Compound That Improves Peripheral Insulin Sensitivity in Mice Models

**DOI:** 10.1371/journal.pone.0103474

**Published:** 2014-07-18

**Authors:** The PLOS ONE Staff

The fourth author's name is spelled incorrectly. The correct name is: Vijayalakshmi Ranjithkumar. The correct citation is: Anthony J, Kelkar A, Wilankar C, Ranjithkumar V, Bhumra SK, et al. (2013) Discovery of P1736, a Novel Antidiabetic Compound That Improves Peripheral Insulin Sensitivity in Mice Models. PLoS ONE 8(10): e77946. doi:10.1371/journal.pone.0077946
